# Acute Distress Respiratory Syndrome After Subarachnoid Hemorrhage: Incidence and Impact on the Outcome in a Large Multicenter, Retrospective Cohort

**DOI:** 10.1007/s12028-020-01115-x

**Published:** 2020-10-20

**Authors:** Aurélien Mazeraud, Chiara Robba, Paola Rebora, Carolina Iaquaniello, Alessia Vargiolu, Verena Rass, Elisa Gouvea Bogossian, Raimund Helbok, Fabio Silvio Taccone, Giuseppe Citerio

**Affiliations:** 1 Neurointensive Care Unit, GHU Paris - Psychiatrie et neurosciences, rue Cabanis 1, 75014 Paris, France; 2 School of Medicine and Surgery, University of Milano - Bicocca, Via Cadore 48, 20900 Monza, Italy; 3 Department of Anaesthesia and Intensive Care, Policlinico San Martino IRCCS for Oncology and Neuroscience, Largo R.Benzi 10, 16132 Genova, Italy; 4 Department of Neurology, Neurological Intensive Care Unit, Medical University of Innsbruck, Anichstrasse 35, 6020 Innsbruck, Austria; 5 Department of Intensive Care, Erasme Hospital, Université Libre de Bruxelles, Route de Lennik 808, 1070 Brussels, Belgium; 6 Neurointensive Care Unit, San Gerardo Hospital, ASST-Monza, Via G. B. Pergolesi 33, 20835 Monza, Italy

**Keywords:** Acute respiratory distress syndrome, Mechanical ventilation, Subarachnoid hemorrhage

## Abstract

**Background:**

Respiratory complications are frequently reported after aneurismal subarachnoid hemorrhage (aSAH), even if their association with outcome remains controversial. Acute respiratory distress syndrome (ARDS) is one of the most severe pulmonary complications after aSAH, with a reported incidence ranging from 11 to 50%. This study aims to assess in a large cohort of aSAH patients, during the first week after an intensive care unit (ICU) admission, the incidence of ARDS defined according to the Berlin criteria and its effect on outcome.

**Methods:**

This is a multicentric, retrospective cohort study in 3 European intensive care units. We collected data between January 2009 and December 2017. We included adult patients (≥ 18 years) with a diagnosis of aSAH admitted to the ICU.

**Results:**

A total of 855 patients fulfilled the inclusion criteria. ARDS was assessable in 851 patients. The cumulative incidence of ARDS was 2.2% on the first day since ICU admission, 3.2% on day three, and 3.6% on day seven. At the univariate analysis, ARDS was associated with a poor outcome (*p* = 0.005) at ICU discharge, and at the multivariable analysis, patients with ARDS showed a worse neurological outcome (Odds ratio = 3.00, 95% confidence interval 1.16–7.72; *p* = 0.023).

**Conclusions:**

ARDS has a low incidence in the first 7 days of ICU stay after aSAH, but it is associated with worse outcome.

**Electronic supplementary material:**

The online version of this article (10.1007/s12028-020-01115-x) contains supplementary material, which is available to authorized users.

## Introduction

Aneurismal subarachnoid hemorrhage (aSAH) affects 9/100,000 persons per year worldwide. The mortality rate due to aSAH is between 35 and 40%, and the disability rate among aSAH survivors reaches 50% [1]. This overall poor prognosis is influenced by several complications such as rebleeding and delayed cerebral ischemia and medical complications during the intensive care unit (ICU) stay. Pulmonary derangements, mainly pneumonia, and acute respiratory distress syndrome (ARDS) are the most frequent ICU complications in aSAH patients. Historical data describe an incidence of ARDS in patients admitted to the ICU for aSAH ranging from 11 to 50% [2–5]. A recent clinical study found that ARDS was independently associated with mortality [2], but its association with functional outcome remains controversial [3, 6]. The heterogeneity of results could be due to the lack of a standard definition and to the differences in treatment protocols among centers, e.g., in the ventilation policies. The Berlin Criteria [7], an updated definition for ARDS, aimed to simplify and standardize its diagnosis, have never been applied to the aSAH population.

Therefore, we conducted a multicenter, retrospective, international study, including a large cohort of patients with aSAH, aiming to assess the incidence of ARDS as defined by the Berlin criteria [7] during the first week after ICU admission and its association with patients' outcomes.

## Methods

### Study Cohort

**Fig. 1:**
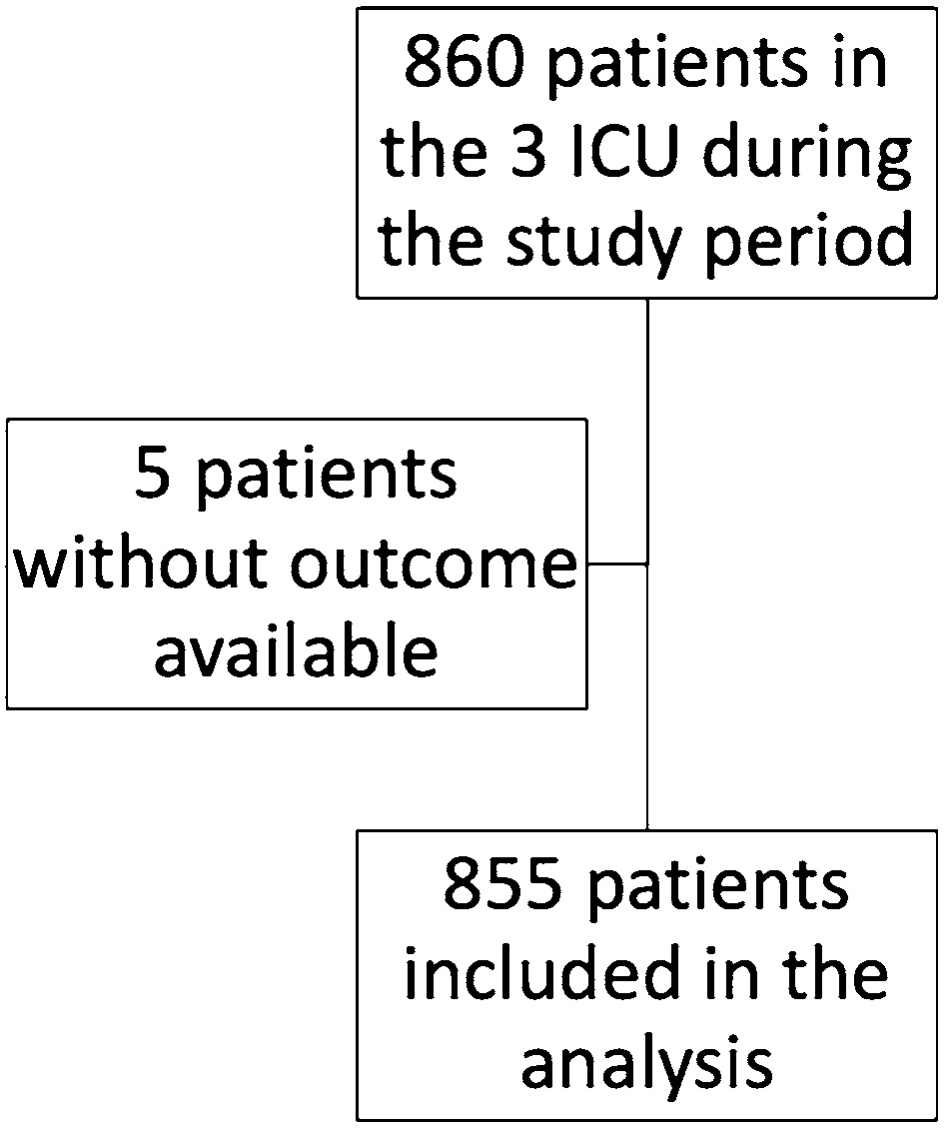
Flow-chart of patients included

This multicenter retrospective study includes consecutive aSAH patients admitted to the neuro-intensive care unit (NICU) of the "San Gerardo" Hospital in Monza, the NICU of the "Medical University Hospital" of Innsbruck and the medical-surgical ICU of the "Erasme Hospital (ULB)" in Brussels between January 2009 and December 2017 (Fig. 1).

Inclusion criteria were:

- Age ≥ 18 years,
- A diagnosis of aSAH confirmed by computed tomography (CT) scan and by an angio-CT or angiography,
- Admission to an ICU involved in the study,
- Presence of information regarding the outcome at ICU discharge.

Exclusion criteria were:

- Age < 18 years.
- Non-aneurysmal SAH.
- No available data on ICU outcome.

### Aims of the Study

The aims of this study were:

- To assess the incidence of ARDS, according to the Berlin Criteria during the first week from ICU admission,
- To evaluate the association between ARDS and neurological outcome at ICU discharge.

### Data Collection

- *Patients' characteristics*

We used the electronic data systems of each center to collect data regarding the patients' previous medical history, including age, smoking habits, and pre-injury comorbidities (e.g., hypertension and chronic obstructive pulmonary disease history). We assessed the Glasgow Coma Scale score (GCS) [8] on admission and the World Federation of Neurosurgical Societies grading system for aSAH (WFNS) [9] for each patient according to their medical records. We recorded the modified Fisher scale (mFisher) [10], the arteriography or angio-CT findings (such as aneurysm location), the information regarding the aneurysm treatment (if surgical or endovascular procedure), and we noted whether and how the invasive intracranial pressure was monitored (through an external ventricular drain (EVD) or an intraparenchymal catheter). Neurological outcome was assessed through the Glasgow Outcome Score (GOS) [11] or the modified Rankin scale (mRS) [12] at ICU discharge. To homogenize the outcome evaluation, we converted the mRS score to GOS score. Thus, a mRS of 0 or 1 corresponds to a GOS score of 5, a mRS of 2 to a GOS score of 4, mRS 3 or 4 to a GOS of 3, mRS 5 to a GOS of 2 and mRS 6 to a GOS score of 1. We then defined a good outcome as a GOS score of 4 or 5. We defined an unfavorable outcome as a GOS ≤ 3.

We recorded the length of mechanical ventilation in hours and ICU length of stay (LOS) in days and defined prolonged mechanical ventilation as > 7 days.

- *Ventilation and oxygenation data*

For each patient, we collected data regarding oxygenation, ventilator settings (such as tidal volume (TV), positive end-expiratory pressure (PEEP)), and arterial blood gas analysis (ABG): pH, partial pressure in oxygen (PaO_2_), and partial pressure of carbon dioxide (PaCO_2_) at day one, three and seven from ICU admission. We collected the lowest and highest value for each variable at each daily time point, then we calculated the lowest values of the ratio of the PaO_2_ over the inhaled fraction of oxygen (PaO_2_/FiO_2_). We defined a PaO_2_ value < 60 mmHg as the cut-off for hypoxemia.

Two trained investigators for each center reviewed all chest X-rays independently, checking for the presence of bilateral infiltrates. In case of disagreement, a third investigator was involved in interpreting the radiological findings.

To evaluate the ARDS incidence in our cohort, we applied the Berlin definition of ARDS that includes:

- Onset within one week of a known clinical insult (here aSAH),
- the presence of bilateral chest opacities on X-Ray, not entirely explained by effusion, atelectasis, or cardiac failure,
- the presence of a PaO_2_/FiO_2_ ratio ≤ 300 and
- a PEEP level of 5 cmH_2_O [7].

Regarding the imaging criteria, every case was reviewed in light of echocardiography, electrocardiography when available, or the medical file to rule out the presence of cardiac failure. When the ARDS criteria were fulfilled, we assessed whether the same episode lasted for multiple days during the first week of ICU admission, thus avoiding the risk of a repeated diagnosis.

### Statistical Analysis

We described quantitative variables as the median and interquartile range (IQR), and categorical variables as number and percentage. In order to compare the characteristics of patients with or without ARDS at ICU admission, and with favorable (GOS 4–5), or unfavorable (GOS 1–3) primary outcome, we computed differences between medians (and bootstrap confidence intervals) and proportions (and exact confidence intervals with Yates’ continuity correction) for continuous and categorical data, respectively.

We estimated the incidence of ARDS, as defined by the Berlin definition, by Aalen–Johansen estimator accounting for competing events of death and discharge and plotted stacked crude cumulative incidence by time since ICU admission. We then conducted a generalized linear mixed model with logit link in order to adjust for the variables affecting the neurological outcome. We included all variables that presented an association with the outcome with a *p* value < 0.15 (by Fisher exact and Wilcoxon test for categorical and continuous variables, respectively) excluding collinear variables [13]. In order to account for center effect, we included a random effect of center in the model. The linearity of continuous variables was checked by the use of polynomials and variables not fulfilling the assumption were categorized for ease of interpretation. Due to missing values in predictors (age 1%, high blood pressure 0.8%, EVD 0.8%, Glasgow coma score at admission 1%, WFNS score 0.9%, mFisher 0.9%, Surgery 1%, white blood count at admission 15.9%, highest temperature at day 1 0.7%), both complete case analysis and multiple imputations were performed. We assumed data were missing at random, in particular for white blood count at admission, that presented the highest missing rate, we believed missing was mainly due to underreporting values from peripheral centers addressing the patient and that white blood count at day 3 and 7 would well allow to impute its value. We performed multiple imputation by MICE algorithm with the method of the chained equation. Ten imputed datasets were created using the following variables: age, ARDS occurrence, EVD, admission GCS, WFNS, mFISHER, surgery, WBC at admission and at day 3 and 7, hypoxia, prolonged mechanical ventilation, smoking history, chronic obstructive pulmonary disease, high blood pressure, aspiration pneumonia, intracranial pressure monitoring, use of neuromuscular blockers, the highest temperature at admission, pathological bronchial secretions, bilateral chest infiltrates, mean arterial pressure at admission, procalcitonin at admission, C reactive protein at admission, heart rate at ICU admission, ICU length of stay and outcomes. Results are presented as odds ratios (OR) with a 95% confidence interval (CI).

All tests were two-sided at a 0.05 significance level; we performed the statistical analysis using R, software version 3.5.2 [14].

## Results

A total of 855 patients fulfilled the inclusion criteria; 851 patients were assessable for ARDS occurrence and included in the analysis (Fig. 1). Table 1 shows the main characteristics of our cohort. Most of the patients were female (59.3%), with a median age of 56 years [47–67]. At day one, three, and seven from ICU admission, 61.8% (528/851), 58.9% (379/643), and 58.6% (301/514) of patients were receiving mechanical, respectively.

**Table 1:** General baseline characteristics of patients with a diagnosis of subarachnoid hemorrhage, in the presence of ARDS within 7 days from ICU admission

	Overall	Patients without ARDS	Patients with ARDS	Difference with CI95%	Missing
n	851	820 (96.4%)	31 (3.6%)		%
Age years (median [IQR])	56 [47, 67]	56 [47, 67]	59 [49, 67]	2.5 (4.2, 9.5)	1.0
Male *n* (%)	345 (40.7)	329 (40.2)	16 (53.3)	13.1 (− 6.7,33.0)	0.9
Smoking history *n* (%)	213 (25.3)	209 (25.8)	4 (13.3)	− 12.4 (− 26.7,1.8)	1.7
COPD *n* (%)	43 (5.1)	40 (4.9)	3 (10.0)	5.1 (− 7.5–17.6)	1.6
High blood pressure *n* (%)	377 (44.7)	361 (44.3)	16 (53.3)	9.0 (− 10.9,28.9)	1.4
Chronic kidney failure *n* (%)	31 (3.7)	29 (3.6)	2 (6.7)	3.1 (− 7.6, 13.8)	1.6
Re-bleeding *n* (%)	82 (9.7)	80 (9.8)	2 (6.7)	3.1 (− 14.0, 7.8)	1.0
Glasgow motor score at admission (median [IQR])	6 [2, 6]	6 [2, 6]	5 [2, 6]	− 1 (− 3, 0)	1.3
Glasgow coma score at admission (median [IQR])	14 [5, 15]	14 [5, 15]	7 [4, 14]	− 7 (− 8,0.5)	1.0
WFNS score (median [IQR])	2 [1, 5]	2 [1, 5]	4 [2, 5]	2 (1, 3)	0.9
mFisher (median [IQR])	4 [3, 4]	4 [3, 4]	4 [4]	0 (0,0)	0.9
Posterior circulation *n* (%)	148 (17.5)	145 (17.7)	3 (10.0)	− 7.7 (− 5, 20.5)	0.9
Endovascular treatment *n* (%)	405 (47.8)	391 (47.9)	14 (46.7)	− 1.2 (− 20.1, 18.2)	1.0
Surgery *n* (%)	274 (32.3)	257 (31.5)	17 (56.7)	25.2 (5.5, 45.0)	1.0
ICP catheter insertion *n* (%)	268 (31.6)	246 (30.0)	22 (73.3)	43.3 (25.4,61.2)	0.8
EVD insertion *n* (%)	369 (43.4)	346 (42.2)	23 (76.7)	32.0 (14.6,49.4)	0.8

*ARDS* acute respiratory distress syndrome, *COPD* chronic obstructive pulmonary lung disease, *EVD* external ventricular drain, *ICP* intracranial pressure, *IQR* Interquartile range, *mFisher* modified Fisher scale, *WFNS* World Federation of Neurosurgical Societies grading system for aSAH

### Incidence of ARDS According to Berlin Criteria

**Fig. 2:**
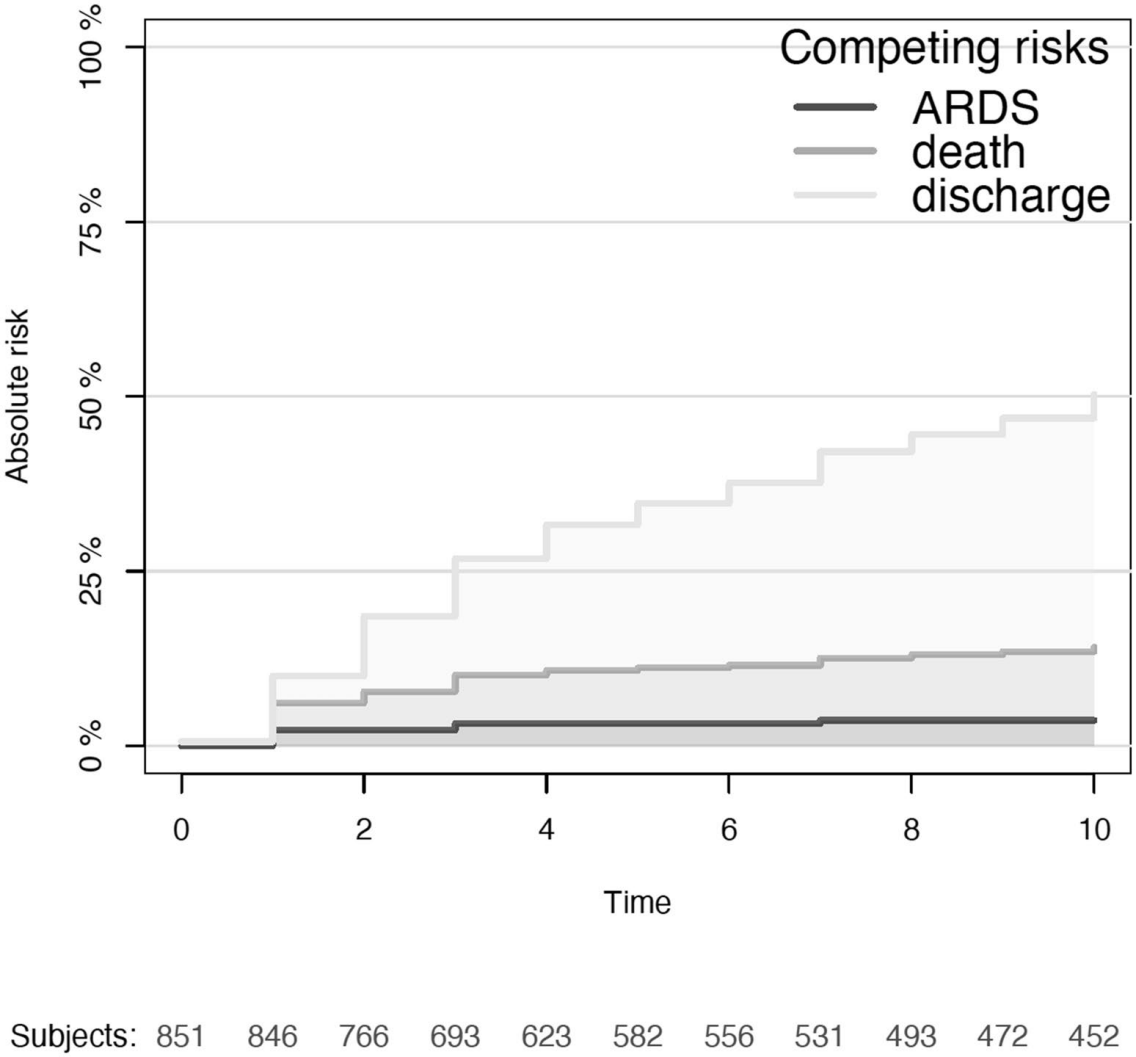
Stacked curves of crude incidence of the competing events ARDS, mortality and discharge by time since ICU admission. The dark grey area represents ARDS incidence, light grey area mortality and light-light grey discharge. For example, among the 851 patients included, at day 3 3.2% had developed ARDS, 6.9% were dead (in ICU without ARDS) and 16.7% had been discharged (without ARDS)

The cumulative incidence of ARDS was 2.2% (95%CI 1.2; 3.2%) at day one, 3.2%(95%CI 2.2;4.4%) at day three, and 3.6% (95%CI 2.4;4.9%) at day seven (Fig. 2). Individual ARDS criteria are depicted in the supplementary table. 19 (61.3%) out of the 31 patients presenting an ARDS within 7 days after ICU admission had transthoracic echocardiography, 8/19 (42.1%) patients presented an abnormal ejection fraction. In these patients, the pulmonary opacities were considered incompletely explained by the occurrence of cardiac diastolic failure.

Patients who presented ARDS were similar for demographic characteristics (including hypertension and smoke) but presented with a more severe aSAH, as for neurological status at admission, according to the GCS, and neuro-radiological findings (mFisher) (Table 1). We did not find any difference in aneurysms' location, but patients with ARDS underwent more frequently invasive procedures such as aneurysm clipping, EVD positioning, and ICP catheter insertion (Table 1).

In the whole cohort, the lowest PaO_2_/FiO_2_ ratio in the first week was 230 [168, 305], 234 [173, 311] in patients without ARDS and 150 [118, 179] in patients who developed ARDS (*p* < 0.0001, Table 2). Patients without ARDS were ventilated with lower TV (420 mL [400, 460] vs. 460 mL [410, 500], *p* = 0.099, Table 2), and neuromuscular blockade was less frequently used (8 (26.7%) vs. 121 (15.2%), *p* = 0.15, Table 2).

**Table 2:** Characteristics of patients with a diagnosis of subarachnoid hemorrhage according to the presence or absence of a concomitant acute respiratory distress syndrome

	Overall	No ARDS	ARDS	Difference estimates with IC95%	Missing
*n*	851	820	31		
Bilateral chest infiltrates	38 (4.5)	7 (0.9)	31 (100.0)		0.0
Highest temperature ˚C (median [IQR])	37.7 [37.2, 38.3]	37.7 [37.2, 38.3]	37.7 [37.1, 38.1]	0 (− 0.5, 0.4)	1.3
Purulent secretions *n* (%)	279 (32.9)	265 (32.2)	14 (45.2)	13.0 (− 6.7, 32.3)	0.0
White blood count day 0, as G/L (median [IQR])	11.6 [9.1, 14.4]	11.5 [9.1, 14.2]	12.6 [10.0, 17.6]	1.1 (− 1.1, 4.1)	15.9
Highest PaO_2_/FiO_2_ ratio (median [IQR])	411 [346, 474]	413 [349, 475]	372 [254, 444]	− 41 (− 129, 11)	14.2
Lowest PaO_2_/FiO_2_ ratio (median [IQR])	230 [168, 305]	234 [173, 311]	150 [118, 179]	− 84 (− 109, − 57)	14.2
Neuromuscular blockage (%)	129 (15.3)	121 (14.9)	8 (26.7)	10.9 (− 6.3, 28.2)	4.1
Highest PEEP cm H_2_O (median [IQR])	8 [5, 10]	8 [5, 9]	10 [7, 10]	2 (0,3)	32.4
Highest tidal volume mL (median [IQR])	520 [481, 599]	524 [487, 600]	487 [420, 510]	− 37 (− 73,− 20)	37.7
Lowest tidal volume mL (median [IQR])	460 [410, 500]	460 [410, 500]	420 [400, 460]	− 40 (− 50, 20)	41.5
Highest PaO_2_ mmHg (median [IQR])	127 [103, 180]	127 [103, 178]	168 [125, 209]	35 (− 3, 62)	0.0
Lowest PaO_2_ mmHg (median [IQR])	80 [70, 98]	80 [70, 99]	74 [63, 80]	− 6 (− 10, 1)	0.0
Prolonged ventilation (7 days) *n* (%)	302 (35.2)	287 (35.0)	15 (48.4)	13.4 (− 6.2, 33.0)	0.6
Ventilation Time (median [IQR])	2 [0, 11]	2 [0, 11]	6 [2, 15]	− 3 (− 6, 1)	0.6
ICU length of stay days (median [IQR])	11 [4, 21]	11 [4, 21]	9 [2, 17]	− 2 (− 8, 5)	0.6
Glasgow outcome scale 1	167 (19.6)	150 (18.3)	17 (54.8)	36.5 (17.2, 55.9)	0
2	77 (9.01)	74 (9.0)	3 (9.7)	0.7 (10.6, 11.9)	
3	96 (11.3)	93 (11.3)	3 (9.7)	− 1.6 (− 14.0, 10.6)	
4	115 (13.5)	110 (13.4)	5 (16.1)	2.7 (12.1, 17.5)	
5	396 (46.5)	393 (47.9)	3 (9.7)	− 3 (− 50.9, 25.6)	
Adverse outcome *n* (%)	340 (40.0)	317 (38.7)	23 (74.2)	35.5 (18.1, 53.0)	0.0

Highest and lowest values within 7 days were collected

*ICU* intensive care unit ,*IQR* interquartile range, *PaO*_*2*_/*FiO*_*2*_ the ratio between the arterial partial pressure in oxygen (PaO_2_) in mmHg and the inhaled fraction of oxygen (FiO_2_), *SD* standard deviation

### Association Between ARDS and Patients' Outcome

Overall, 342 patients (40.0%) presented a poor neurological outcome at ICU discharge (Fig. 2), 23 (74.2%) among patients with ARDS, and 319 (38.7%), among others (*p* = 0.005). Patients with ARDS had a higher duration of mechanical ventilation (6 [2, 15] vs. 2 days [0, 11], *p* = 0.002), but a similar LOS (9 days [2, 17] vs. 11 days [4, 21], *p* = 0.198, Tables 2, 3).

**Table 3:** Outcomes of patients with a diagnosis of subarachnoid hemorrhage admitted to the intensive care unit

	Overall	Good outcome	Bad outcome	Difference estimates with IC95%	Missing
*n* (%)	851	511 (60.0)	340 (40.0)		(%)
Age years (median [IQR])	56 [47, 67]	54 [46, 62]	62 [51, 72]	8 (5,11)	1.0
Male *n* (%)	345 (40.7)	218 (42.8)	127 (37.5)	− 5.4 (− 12.3,1.6)	0.9
Smoking History *n* (%)	213 (25.3)	128 (25.1)	85 (25.6)	0.5 (− 5.8,6.7)	1.7
COPD *n* (%)	43 (5.1)	23 (4.5)	20 (6.0)	1.5 (1.9,4.9)	1.6
High blood pressure *n* (%)	377 (44.7)	205 (40.3)	172 (51.3)	11.0 (4.0,18.2)	1.4
Chronic kidney failure *n* (%)	31 (3.7)	12 (2.4)	19 (5.7)	3.3 (0.3,6.4)	1.6
Re-bleeding *n* (%)	82 (9.7)	38 (7.5)	44 (13.0)	5.5 (1.1,10.1)	1.0
Glasgow motor score at admission (median [IQR])	6 [2, 6]	6 [6]	4 [1, 6]	− 2 (− 4,− 2)	1.3
Glasgow coma scale at admission (median [IQR])	14 [5, 15]	15 [13, 15]	6 [3, 14]	− 9 (− 10,− 7)	1.0
WFNS (median [IQR])	2 [1, 5]	1 [1, 3]	5 [2, 5]	4 (2,4)	0.9
mFISHER (median [IQR])	4 [3, 4]	3 [2, 4]	4 [4]	1 (0,1)	0.9
Posterior circulation *n* (%)	148 (17.5)	85 (16.7)	63 (18.6)	1.9 (− 3.6,7.4)	0.9
Endovascular treatment *n* (%)	405 (47.8)	255 (50.1)	150 (44.4)	− 5.7 (− 12.8,1.4)	1.0
Surgery *n* (%)	274 (32.3)	128 (25.1)	146 (43.2)	18.1 (11.3,24.8)	1.0
ICP catheter *n* (%)	268 (31.6)	105 (20.6)	163 (48.1)	27.5 (20.9,34.1)	0.8
EVD insertion *n* (%)	369 (43.4)	167 (32.7)	202 (59.4)	26.7 (19.9,33.6)	0.8
Aspiration pneumonia *n* (%)	57 (6.7)	18 (3.5)	39 (11.5)	8.0 (4.0,12.0)	0.9
ARDS diagnosis *n* (%)	31 (3.6)	8 (1.6)	23 (6.8)	5.2 (2.1,8.3)	0.0
Bilateral chest infiltrates *n* (%)	38 (4.5)	10 (2.0)	28 (8.2)	6.3 (2.9,9.7)	0.0
Highest temperature °C (median [IQR])	37.7 [37.2, 38.3]	37.6 [37.1, 38.1]	37.9 [37.3, 38.5]	0.3 (0.1,0.4)	1.3
Purulent secretions *n* (%)	279 (32.9)	95 (18.6)	184 (54.1)	35 (29,42)	0.0
White blood cell count day 1 (median [IQR])	11.6 [9.1, 14.4]	11.2 [9.0, 13.7]	12.5 [9.4, 15.7]	1.3 (0.2,1.8)	15.9
Highest PaO_2_/FiO_2_ ratio (median [IQR])	411 [346, 474]	409 [346, 473]	414 [345, 474]	5 (− 12,21)	14.2
Lowest PaO_2_/FiO_2_ ratio (median [IQR])	230 [168, 305]	251 [181, 328]	212 [158, 265]	− 39 (− 55,− 18)	14.2
Neuromuscular blockage *n* (%)	129 (15.3)	49 (9.6)	81 (23.8)	14.2 (8.8,19.7)	4.1
Highest PEEP cm H_2_O (median [IQR])	8 [5, 10]	5 [5, 8]	8 [5, 10]	3 (2,3)	32.4
Highest tidal volume mL (median [IQR])	520 [481, 599]	513 [480, 591]	530 [487, 601]	17 (0,39)	37.7
Lowest tidal volume mL (median [IQR])	460 [410, 500]	475 [427, 503]	450 [400, 494]	− 25 (− 36,10)	41.5
Highest PaO_2_ mmHg (median [IQR])	127 [103, 180]	119 [97, 167]	145 [112, 195]	21 (10,32)	0.0
Lowest PaO_2_ mmHg (median [IQR])	80 [70, 98]	80 [70, 104]	79 [70, 91]	2 (− 2,4)	0.0
Hypoxemia < 60 mmHg *n* (%)	61 (7.1)	33 (6.5)	28 (8.2)	1.7 (− 2.1,5.6)	0.0
Prolonged ventilation (7 days) *n* (%)	302 (35.2)	87 (17.0)	215 (63.2)	46.2 (39.9,52.5)	0.6
Ventilation time in days (median [IQR])	2 [0, 11]	1 [0, 3]	10 [3, 21]	10 (8,11)	0.6
ICU length of stay in days (median [IQR])	11 [4,21]	9 [3, 16]	17 [5, 29]	8 (5,10)	0.6

*COPD* chronic obstructive pulmonary lung disease, *EVD* external ventricular drain, *ICP* intracranial pressure, *mFisher* modified Fisher score, *PaO*_*2*_*/FiO*_*2*_ the ratio between the arterial partial pressure in oxygen (PaO_2_) in mmHg and the inhaled fraction of oxygen (FiO_2_), *WFNS* World Federation of Neurosurgical Societies grading system for aSAH

Several variables were associated with poor ICU outcome (Table 3): older age, history of high blood pressure, rebleeding, worse neurological status at admission (assessed by motor and total GCS, as well as WFNS score), modified Fisher grade, the need for surgical intervention, or insertion of an ICP catheter or EVD, the occurrence of aspiration pneumonia, the temperature at admission, ARDS diagnosis, radiological opacities, the use of neuromuscular blockers, tidal volume and PEEP settings, a high white blood cell (WBC) count, higher PaO_2_ values, higher duration of intubation and LOS.

To perform the multivariable regression analysis, we excluded the collinear variables such as radiological opacities, the use of neuromuscular blockers, tidal volume and PEEP settings. We, therefore, included: age, high blood pressure, admission GCS, WFNS, mFisher, need for ICP or EVD, surgical intervention, WBC at admission, highest temperature, aspiration pneumonia, hypoxemia, ARDS and prolonged mechanical ventilation. After multiple imputation, the multivariable model evidenced that age > 65 years (OR = 3.52, 95%CI = 2.24–5.53, *p* < 0.001), prolonged duration of mechanical ventilation (OR = 4.75, 95% CI = 3.18–7.09, *p* < 0.001), GCS < 8 at admission (OR = 0.47, 95%CI = 0.23–0.93, *p* = 0.033), a Fisher score of 3 or above (OR = 1.76, 95%CI = 1.04–2.95, *p* = 0.033), neurosurgery intervention (OR = 1.70, 95%CI = 1.16–2.50. *p* = 0.0065) and ARDS (OR = 3.00, 95%CI = 1.16–7.72, *p* = 0.023) were associated with an unfavorable outcome at discharge from ICU (Table 4).

**Table 4:** Multivariable model evaluating the risk of an adverse outcome, defined as Glasgow outcome score ≤ 3

	Complete case analysis (*n* = 712, 268 with bad outcome at ICU discharge)				Multiple imputation analysis (n = 851, 340 with bad outcome at ICU discharge)			
	OR	2.5%	97.5%	*p* value	OR	2.5%	97.5%	*p* value
ARDS occurrence	2.6	1.01	6.71	0.048	3.00	1.16	7.72	0.023
Age > 65 versus Age < 50 y	3.16	1.93	5.18	< 0.001	3.52	2.24	5.53	< 0.001
Age 50–65 versus Age < 50 y	1.27	0.8	2.02	0.3043	1.23	0.81	1.88	0.3324
EVD	1.39	0.88	2.19	0.154	1.27	0.84	1.92	0.254
Admission Glasgow Coma Scale 6–8 versus < 6	0.55	0.27	1.12	0.0979	0.58	0.29	1.14	0.1163
Admission Glasgow Coma Scale > 8 versus < 6	0.54	0.25	1.16	0.113	0.47	0.23	0.94	0.033
WFNS (by point)	1.24	1	1.53	0.046	1.21	1.00	1.46	0.055
mFISHER_ ≥ 3 versus < 3	2.15	1.13	4.09	0.020	1.76	1.05	2.95	0.033
Underwent surgery	1.84	1.23	2.75	0.003	1.70	1.16	2.50	0.007
WBC at admission (by G/L)	1.02	0.98	1.07	0.255	1.00	0.96	1.04	0.954
Hypoxia_anytime	1.36	0.66	2.83	0.405	1.28	0.66	2.47	0.469
Need for prolonged mechanical ventilation	4.67	3.02	7.24	< 0.001	4.75	3.18	7.09	< 0.001

*ARDS* acute respiratory distress syndrome, *EVD* external ventricular drain, *WFNS* World Federation of Neurosurgical Societies grading system for aSAH, *mFisher* modified Fisher score, *OR* odds ratio, *WBC* white blood cell count

## Discussion

To the best of our knowledge, this is the most extensive multicenter study to describe ARDS occurrence after aSAH using the Berlin definition in patients admitted to an ICU.

Our main findings are the low incidence of ARDS in this population and, when present, its relevant impact on functional outcome. The large size of our cohort and the inclusion of three different centers are important elements to support the accuracy of ARDS incidence evaluation and its external validity.

We evaluated ARDS secondary to subarachnoid hemorrhage within the first seven days after the ICU admission, at three different time points within the first week. In light of this, our findings could be explained by assuming that in our patients ARDS resolved rapidly, but radiologic improvement is usually slower than clinical improvement. Therefore, assessing patients on three timepoints with chest X-ray is a suitable strategy to evaluate ARDS incidence. Few patients presented bilateral chest infiltrates on day seven without clinical manifestations of ARDS (11 patients representing 1.3% of the population). Also, in non-ARDS patient with P/F < 300, we observed unilateral opacities on chest X-ray in 47/356(13.2%) 55/333(16.5%) and 44/240(18.3%) at day 1, 3 and 7, suggesting that aspiration pneumonia or ventilator acquired pneumonia could be one of the causes of hypoxemia (see supplementary table).

Brain injury is a known factor for lung damage. First, swallowing disorders are frequent in brain-injured patients up to the fact that aspiration pneumonia causes the highest attributable mortality of all medical complications following stroke [15, 16]. However, other causative mechanisms have been proposed, such as abnormal immune patterns, catecholamine, and cytokine storm, both increasing pulmonary vascular pressure and activating the lung immune system, increasing its susceptibility to secondary damage [17, 18].

When compared to previous studies, the strength of this study is the rigorous application of stringent criteria to define ARDS. A retrospective study reported an incidence of PaO_2_/FiO_2_ ratio < 300 in nearly one-third of patients with aSAH (27%) without integrating other ARDS criteria [2]. Other studies found an incidence of ARDS between 4 and 18% after aSAH [5], or brain injury using a different definition [19–23]. Our study suggests a lower incidence of ARDS after aSAH, according to the Berlin definition. This new definition restricts ARDS diagnosis to patients with at least 5 cmH_2_O of PEEP, which could explain the lower incidence found in our study, compared to previous ones. Another reason could be related to the increasing application of protective ventilation strategies in our institutions over the last years [24]. Protective mechanical ventilation includes low tidal volume, adequate PEEP levels, low plateau pressure, and recruitment maneuvers when needed, and it has been associated with reduced mortality in non-ARDS critically ill patients [25, 26].

Although the beneficial effect of protective lung ventilation and respiratory strategies is well established in the general ICU population and the operating room, the application of these techniques in neuro-critical care patients is contrasting [27]. However, recent studies support the use of protective strategies and, in particular, of low driving pressure and low tidal volumes even in brain-injured patients [27].

We observed an increase of three times in the odds of poor neurological outcome in patients with ARDS after adjusting to usual variables (OR = 3.00, 95%, CI 1.16;7.72). Our observation is in line with the effect size of ARDS on mortality observed in other studies approaching a 1.65-fold increase in the risk for mortality [2]. As ARDS is associated with a prolonged ventilation time and a worst initial neurological injury, we could hypothesize that these represent confounding factors linking ARDS to the outcome. When adjusting for these factors, presenting ARDS was associated with worse neurological outcome at ICU discharge. Overall, only 31 patients presented an episode of ARDS. Even with a relatively small occurrence rate of ARDS, we showed an association with the outcome that resisted multiple statistical adjustments. The pathophysiological mechanism linking ARDS to worse neurological outcomes can be multifactorial. Inadequate brain oxygenation conducting to cell hypoxia and neuronal death is one of the main hypotheses. Thus, the recommended target of PaO_2_ in brain-injured patients with healthy lungs is above 75 mmHg, whereas lower PaO_2_ targets (55–80 mmHg) are suggested in patients without brain injury [18, 24].

### Limitations

Several limitations need to be mentioned in this study.

ARDS incidence was evaluated accordingly to the daily worst PaO_2_/FiO_2_ value. Thus, we might have even overestimated the incidence of ARDS, as daily lowest PaO_2_/FiO_2_ could be the result of temporary episodes of hypoxemia, such as those occurring during an atelectasis episode, during the transports to the radiologic suite, or during an angiographic procedure. Also, the participation of cardiogenic edema to ARDS was ruled out according to medical files, transthoracic and EKG interpretation in the medical files.

In our study, we did not investigate the etiology of ARDS. ARDS after aSAH can be multifactorial. Different mechanisms have been described, such as aspiration, neurogenic pulmonary edema, or brain immune system interaction. Surprisingly, the incidence of aspiration pneumonia and the presence of pathological pulmonary secretions did not differ in patients with and without ARDS.

Moreover, we focused on the early outcome after aSAH, at ICU discharge. We considered the time in ICU by a Cox model on ICU mortality and found consistent results (the hazard of ICU mortality in patients with ARDS was higher than patients without ARDS: hazard ratio 2.41, 95%CI: 1.39; 4.18). Many other factors would have influenced the long-term neurological outcome in aSAH, not only ARDS; as e.g., the aneurysm treatment, complications and the rehabilitation phase.

Due to the retrospective observational design of this study, we reported an association but not causality.

The retrospective data collection resulted also in the lack of precise and complete data regarding ventilation and other confounders that might have influenced the patients' outcome.

## Conclusions

Our results demonstrate in an extensive series of aSAH patients that ARDS has a lower incidence than previously described in the first seven-day period of ICU admission. It might be due to a more rigorous definition of ARDS but also to the systematic use of protective ventilation strategies. The most important predictors of outcome at ICU discharge are age, the severity of aSAH, the need for surgery or prolonged mechanical ventilation and ARDS occurrence.

## Electronic supplementary material

Below is the link to the electronic supplementary material.

Supplementary file1 (DOCX 13 kb)

## Data Availability

The datasets analyzed during the current study are available from the corresponding author upon reasonable request.

## Abbreviations

ABG: Arterial blood gas analysis
ARDS: Acute respiratory distress syndrome
aSAH: Aneurysmal subarachnoid hemorrhage
cmH_2_O: Centimeters of water
EVD: External ventricular drain
EKG: Electrocardiogram
FiO_2_: Inhaled oxygen fraction
GCS: Glasgow coma score
GOS: Glasgow outcome score
ICU: Intensive care unit
IQR: Interquartile range
LOS: Length of stay
mFisher: Modified Fisher scale
mmHg: Millimeters of mercury
mRS: Modified Rankin scale
NICU: Neurointensive care unit
NPE: Neurogenic pulmonary edema
PaCO_2_: Arterial partial pressure of carbon dioxide
PaO_2_: Arterial partial pressure of oxygen
PaO_2_/FiO_2_: The ratio of the arterial partial pressure of oxygen over the inhaled fraction of oxygen
PEEP: Positive end-expiratory pressure
SaO_2_: Arterial oxygen saturation
TV: Tidal volume
VAP: Ventilator-associated pneumonia
WFNS: World Federation of Neurosurgical Societies grading system for aSAH
