# Supplementary material for: Acute Distress Respiratory Syndrome After Subarachnoid Hemorrhage: Incidence and Impact on the Outcome in a Large Multicenter, Retrospective Cohort
Source: Neurocrit Care. 2020 Oct 20;34(3):1000–8. doi: 10.1007/s12028-020-01115-x (PMC7575216; doi:10.1007/s12028-020-01115-x)
Supplement: Supplementary file 1 — Supplementary file1 (DOCX 13 kb) [file 12028_2020_1115_MOESM1_ESM.docx]

|  | Day 1 | Day 3 | Day 7 |
| --- | --- | --- | --- |
| PaO_2_/FiO_2_ ratio < 300 n(%) | 356 | 333 | 240 |
| PEEP≥5 cm H_2_0 n(%) | 162/356 (4) | 329/333 (98.8) | 237/240 (98.8) |
| Bilateral alveolo-interstitial infiltrate at chest X-ray n(%) | 4/356 (1.1) | 0/333 (0) | 3/240 (1.3) |
| Unilateral alveolo-interstitial infiltrate at chest X-ray n(%) | 47/356 | 55/333 (16.5) | 44/240 (18.3%) |

Characteristics of patients with a PaO_2_/FiO_2_ ratio bellow 300 during ICU stay regarding the other Berlin criterion. Of note 236 transthoracic echocardiographies have been realized through ICU stay in which a systolic failure was observed in 21(8.9%) patients. PEEP: Positive end expiratory pressure.
